# Performance and Predictive Value of First Trimester Screening Markers for Down Syndrome in Iranian Pregnancies

**Published:** 2018-09

**Authors:** Reza Heidari, Mostafa Akbariqomi, Elaheh Motevaseli, Mir Davood Omrani, Hamid Kooshki, Ahmad Reza Shamshiri, Shilan Shafei, Moloud Absalan, Mohammad Ali Mazlomi, Soraya Saleh Gargari, Gholamreza Tavoosidana

**Affiliations:** 1 Department of Molecular Medicine, School of Advanced Technologies in Medicine, Tehran University of Medical Sciences, Tehran, Iran; 2 Department of Medical Genetics, Faculty of Medicine, Shahid Beheshti University of Medical Sciences, Tehran, Iran; 3 Nanobiotechnology Research Center, Baqiyatallah University of Medical Sciences, Tehran, Iran; 4 Department of Epidemiology and Biostatistics, School of Public Health, Tehran University of Medical Sciences, Tehran, Iran; 5 Department of Medical Biotechnology, School of Advanced Technologies in Medicine, Tehran University of Medical Sciences, Tehran, Iran; 6 Department of Gynecology & Obstetric, Shohada Tajrish Educational Hospital, Shahid Beheshti University of Medical Sciences, Tehran, Iran

**Keywords:** Down Syndrome, First Trimester Screening, Serum Marker, Sonographic Markers

## Abstract

**Objective:** To investigate the performance of first trimester Down syndrome (DS) screening markers in Iranian pregnancies.Although sonographic and serum markers are currently recommended for the first trimester screening of Down syndrome, the screening performance of the markers depends on the race and ethnicity.

**Materials and methods:** A retrospective case-control study using first trimester screening results recorded with the prenatal diagnostic multi-centers in Iran. A total of 6,384 pregnant women were examined from March 2012 to February 2017. Totally 100 Down syndrome cases and 266 matched controls were selected and the maternal characteristics, sonographic and biochemical screening data were collected. Statistical analysis was performed using logistic regression and descriptive statistics. A decision tree model was designed using the chi-squared automatic interaction detection method based on serum markers.

**Results:** For screening of DS pregnancies, PAPP-A (cut-off 0.795 MoM) yielded the highest sensitivity (86%) and NB marker presented highest specificity (96.24%). combination of the biochemical markers PAPP-A and β-hCG (cut-off: 1.55 MoM) showed the highest sensitivity over other combined markers. The decision-tree model based on serum markers improved (91% DR For a 5% FPR) first trimester screening performance.

**Conclusion:** The novel decision-tree model base on serum markers revealed a better predictive value to achieve high sensitivity and specificity of first trimester Down syndrome screening in Iranian population.

## Introduction

In developed countries, prenatal diagnosis of aneuploidy plays an important role in management of a healthy pregnancy, especially in the first trimester (1). In today's families, the trend is toward having healthy and fewer children (2); therefore, early screening tests, especially in the first trimester of pregnancy are considered necessary to ensure the health of the foetus (3). 

Down syndrome (DS; OMIM number 190685) is a genetic disorder caused by the presence of all or part of an extra copy of chromosome 21. It is the most common chromosomal autosomal aneuploidy in live births (4). DS is associated with congenital abnormalities that include intellectual disability (5), physical-behavioural disorders and major or minor abnormalities in the structure or function of organs (6, 7). Currently, DS is the main reason for which families are referred to prenatal counselling centres. The general incidence of DS is 1 in 700-800 live births worldwide, but in Iran its incidence is 1 in 814 live births (8).

Initially, classification of pregnancy into high risk (> 35 years) or low risk for DS screening was solely based on maternal age, because the incidence of DS in the foetuses of older women is greater (9). In 2007, the revised guidelines of the American College of Obstetricians and Gynecologists proposed that screening for DS should be done for all pregnant women, regardless of age (10). Bogart (1987) introduced β-subunit of human chorionic gonadotropin (β-hCG) level as the first serum marker for screening chromosomal abnormalities (11). Bersinger et al. (1994) later identified pregnancy-associated plasma protein A (PAPP-A) as the second serum marker for screening of DS (12). The concentration of PAPP-A and free β-hCG increase during gestation and it has been proven that, in chromosome aneuploidies, the concentration of these markers clearly changes in the first trimester (13). In DS pregnancies, the β-hCG levels increase significantly over healthy gestational levels, whereas the PAPP-A levels in pregnancies with trisomy 21 decrease significantly (14). 

The first characteristic sonographic marker for screening of DS was introduced by Nicolaide (15) and Ville (16) (1992) as the measurement of nuchal translucency (NT) thickness during the first trimester. A pervious study suggests that a NT thickness between 3.5-4.5 mm is accompanied by a majority chance of the presence of trisomy 21 (17). Another sonographic marker proposed for detection of DS is the absence of foetal nasal bone (NB). NB absence is more frequently seen in foetuses with DS (about 70%) in comparison with normal foetuses (1% to 3%) (18).

The combined first-trimester tests (cFTT) for DS screening include the foetal sonographic markers (NT, NB), maternal serum markers (β-hCG, PAPP-A) and maternal age. The detection rate (sensitivity) of cFTT is reported to be about 90% with 5% false positivity (19). In fact, a certified combination test would decrease the cost and stress of testing by avoiding unnecessary invasive procedures. In addition, cFTT requires specific cut-offs for each laboratory and ethnicity for first trimester screening. 

Several studies have reported that racial-ethnic differences have influenced the factors involved in cFTT and could result in inaccurate screenings in multi-ethnic societies (20-22). It is necessary to consider reliable cut-offs for screening markers of normal and DS foetuses according to their racial characteristics in order to achieve better detection rates. The main purposes of the current study were to bridge the gap by: (1) comparing first trimester screening markers between normal and DS pregnancies in the Iranian population; (2) increasing the effectiveness of the DS screening detection rate by decreasing the percentage of false positive results among Iranian mothers; and (3) helping physicians and pregnant women make decisions about invasive diagnostic tests.

## Materials and methods

This was retrospective case-control study on 6,384 pregnant women who were referred for first-trimester screening of DS at major prenatal diagnostic multi-centres in Tehran, Iran, from March 2012 to Feb 2017. A total of 100 women who carried a DS foetus were identified after confirmation by chorionic villus sampling (CVS) or amniocentesis. At the same time, 266 healthy pregnant women experiencing no medical complications during pregnancy and who delivered normal neonates after follow-up formed the control group. The members of the control group were matched with those in the case group according to body mass index, gestational age and singleton gestation. Exclusion criteria were multiple and IVF pregnancies, invalid biochemical marker levels, smoking and have been diagnosed with diabetes. The study was approved by the ethical committee of Tehran University of Medical Sciences (Ethics code: IR.TUMS.REC.1394.1156) and All pregnant women provided written informed consent prior to entering the study.

The maternal demographic characteristics, sonographic and biochemistry screening results of all participants at 11 + 0 and 13 + 6 weeks of gestation were recorded in electronic databases. NT thickness measurement and NB assessment were performed using transabdominal ultrasound examination based on the reliable guidelines of the Foetal Medicine Foundation. The serum levels of β-hCG and PAPP-A were evaluated by immunofluorescence (Delfia Express System; Perkins-Elmer; USA). To correct for errors caused by race, gestational age, height, mode of conception and weight, the serum marker levels were converted to multiples of the median (MoM). 

Comparisons of the demographic and clinical characteristics in the case and control groups were done using the Mann-Whitney U test for continuous data and the χ^2^ test for categorical data. The maximum Youden J index was selected as the optimal cut-off point in order to obtain the highest sensitivity and specificity (23). A decision tree model was designed using the chi-squared automatic interaction detection (CHAID) method based on serum markers to obtain the best outcome for first trimester screening. In the software settings, the following options were considered: (1) minimum number cases in a parent node of 50 samples and in a child node of 20 samples; (2) adjustment of the misclassification cost of false negative to five times that of a false positive to increase the sensitivity of the decision model; and (3) cross-validation by 20% samples to validate the decision tree model. A p-value ≤ 0.05 was considered statistically significant. All statistical analyses were performed with SPSS for Windows (ver. 24; IBM; USA).

## Results

In this study, 366 pregnant women who met the inclusion criteria were assessed. The clinical and demographic characteristics of the study groups are shown in Table 1. The mean maternal and paternal age of foetus with Down syndrome was 35.8 ± 5.84 and 39.1 ± 6.6 years, respectively. The mean maternal weight of DS foetuses during the first trimester of pregnancy was 73.31 ± 11.88 (SD) kg. There was significant difference in maternal weight and parents age between the DS and normal groups (O< 0.001). 

**Table 1:** clinical and demographic characteristics of the study groups

**Category**	**DS (n: 100)**	**Normal (n: 266)**
Maternal age (Mean ± SD)	35.8 ± 5.84	31.05 ± 4.1
< 35 yrs.	40%	84.58%
≥ 35 yrs.	60%	15.42%
Paternal age (Mean ± SD)	39.1 ± 6.6	33.02 ± 4.5
< 35 yrs.	25%	66.2
≥ 35 yrs.	75%	33.8
Maternal Weight (Kg, Mean ± SD)	73.31 ± 11.88	68.39 ± 12.15
Education of Mother (%)		
Elementary	5%	2%
Junior high school	13%	9%
High school	39%	26%
University	43%	63%
Place of residence (%)		
Urban	41%	71.5%
Rural	59%	28.5%
Sex of Infant (Boy-Girl)	(48%-52%)	(49%-51%)
Family history of aneuploidy(NO-Yes)	(92%-8%)	NO
Diagnostic test (%)		
Karyotype	71%	-_
MLPA	6%	-_
qf-PCR or FISH	23%	-_
NB (Present – Absent, %)	(30% - 70%)	(100% - 0)
Serum PAPP-A, MoM (cI 95%)	0.54(0.5042 - 0.5838 )	1.59(1.22 - 2.1470)
Serum free β-hCG, MoM (cI 95%)	1.68(1.5437 - 1.8191)	1.0807(1.0026 - 1.1542)
NT, mm (cI 95%)	2.64(2.4755 - 2.8420)	1.61 (1.5671 - 1.6581)

DS: Down syndrome; MLPA: Multiplex ligation-dependent probe amplification;qf-PCR:Quantitative fluorescent polymerase chain reaction; FISH: Fluorescent in situ hybridization; NB: Nasal bone; PAPP-A: Pregnancy associated plasma protein A; NT: Nuchal translucency; β-hCG: β human chorionic gonadotrophin

**Table 2:** The efficacy of single and combined markers in DS screening

**Marker**	**Sensitivity (%)**	**Specificity (%)**	**Accuracy (%)**	**PPV (%)**	**NPV (%)**	**LR+**	**LR-**
Single marker (Cut-0ff)							
PAPP-A (0.795 MoM)	86	79.32	81.15	60.99	93.78	4.15	0.17
β-hCG (1.55 MoM)	80	65.41	69.4	46.51	89.69	2.31	0.3
NT (1.89 mm)	77	76.32	76.5	55	89.82	3.25	0.3
NB	70	96.24	91.8	100	89.86	+infinity	0.25
Double marker							
PAPP-A+NT	71	95.86	89.07	86.59	89.79	17.16	0.3
PAPP-A+β-hCG	76	98.5	92.35	95	91.61	50.54	0.24
PAPP-A+NB	64	100	90.16	100	88.08	+infinity	0.36
NT+NB	57	100	88.25	100	88.25	+infinity	0.43
Triple markers							
PAPP-A+β-hCG+NT	60	94.74	85.25	81.08	86.3	11.4	0.42
PAPP-A+NT+NB	52	100	86.89	100	84.71	+infinity	0.48
β-hCG+NT+NB	44	100	84.7	100	82.61	+infinity	0.56
Quadruple markers							
PAPP-A+NB+β-hCG+NT	41	100	83.88	100	81.85	+infinity	0.59

PPV: Positive predictive value; NPV: Negative predictive value; LR: Likelihood ratio

Most of the DS parents lived in rural area and had academic education. As shown, karyotype has mainly been used as a confirmatory test for trisomy 21 diagnosis. The nasal bone was absent in 70% of DS foetuses. In DS group, the median MoM of maternal serum PAPP-A was 0.54 and free β-hCG was 1.68 and the median NT measurement was 2.64 mm. Overall, there was statistically significant difference between the groups for all factors (p < 0.001) except the foetal gender (p > 0.05).

Table 2 shows the efficacy of single and combined markers in DS screening. The observed detection rates at a 5% false positive rate were 77% for NT (cut-off: 1.89 mm), 80% for β-hCG (cut-off: 1.55 MoM), 86% for PAPP-A (cut-off: 0.795 MoM) and 60% for a combination of NT, β-hCG and PAPP-A. The use of combined markers increased the specificity to 100% but decreased the sensitivity to 41%. The combination of the biochemical markers PAPP-A and β-hCG showed the highest sensitivity over other combined markers.

**Figure 1:**
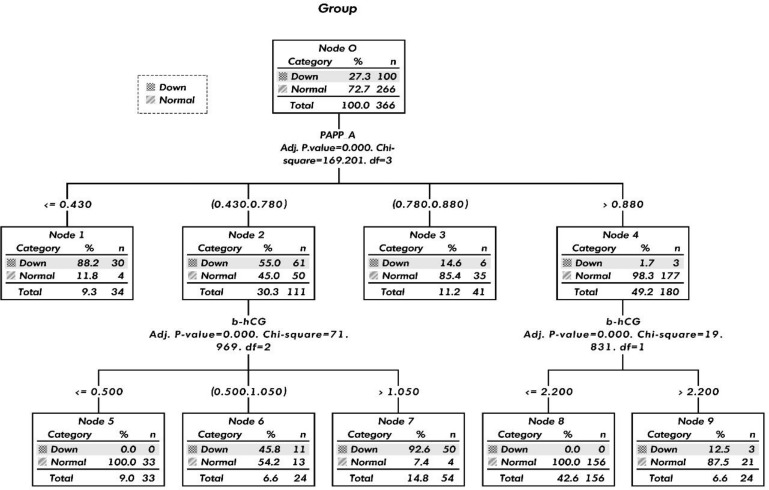
The decision-tree model base on serum markers for a better predictive value detection of Down syndrome

Figure 1 shows that the 366 samples were divided into four main nodes based on the different cut-off levels of the PAPP-A marker (p < 0.001). The decision tree analysis show that the probability having a DS pregnancy of ≤ 0.43 MoM (Node 1) was 88.2%, for the 0.43-0.78 MoM range (Node 2) was 55% for DS and for a normal pregnancy was 45%. By combining the 0.43-0.78 MoM level for PAPP-A (Node 2) with the ≤ 0.5 MoM (Node 5) and > 1.05 MoM (Node 7) for β-hCG, It was shown that all pregnancies were normal in node 5 and 92.6% were DS pregnancies in node 7. The decision tree model provided a sensitivity of 91%, specificity of 92.1% and accuracy of 91.8% for screening of DS in the first trimester.

## Discussion

Given the inconsistency of universal DS screening marker results and pregnancy outcomes in the Iranian population, it seemed necessary to research the national cut-offs for these screening markers to find those that exhibit high efficiency and reliability. The performance of first trimester DS screening markers were evaluated for the case group including 100 DS pregnancies and the 266 matched controls among pregnant Iranian women.

In this study, a decision tree algorithm was used to generate a screening suggestion with greater sensitivity, specificity and accuracy in the first trimester of pregnancy. All of the samples were classified into four groups based on PAPP-A marker levels. It was shown that the highest and the lowest numbers for DS foetuses were in node 2 (PAPP-A values of < 0.43 MoM) and node 4 (PAPP-A values of > 0.88 MoM), respectively. This algorithm showed that the combination of node 2 with β-hCG led to the best detection rate for DS foetuses at > 1.05 MoM (Node 7) and normal foetuses at < 0.5 MoM (Node 5).

Although advanced maternal age is clearly a risk factor for DS, the effect of paternal age on DS incidence remains controversial. Three conflicting opinions exist about the relationship of paternal age and the risk of DS.

A study has reported no correlation between paternal age and risk of DS (20), other study has reported that paternal age increased the risk of DS if the maternal age was over 35 years (21). Recent study has demonstrated that the risk of DS was higher in younger fathers than older fathers (22). In the current study, it was found that advanced paternal and maternal age were significantly (p < 0.001) related to an increased risk of DS. This result is similar to that of Fisch et al. (2003) (24).

The results of the current study revealed that mother’s residential area could also play a role in the incidence of DS. Different factors could be involved in this issue, including nutritional deficiency and chemical and biological contamination. in developing countries, the high usage of fertilizers and pesticides are potential reasons for the elevated risk of DS. In this study, the DS incidence in rural areas was significantly higher than in urban areas These findings are consistent with the results of previous study (25).

NB absence is an important sonographic marker for identifying DS foetuses in singleton pregnancies. NB assessment is a useful method of improving the sensitivity of DS screening and can reduce the false positive rate and the necessity of invasive diagnosis tests (26). The prevalence of NB absence in previous studies differed from that of trisomy 21 (27-29). The inconsistency of these results could be due to maternal ethnicity or an inadequate number of DS samples. The current study showed NB absence in 70% of DS foetuses in the Iranian population, which was similar to that of Italian population as found by Zoppi et al. (2003) (30).

In many countries, all pregnant women are recommended to undergo NT assessment during the first trimester because it can be detect approximately 70% of DS cases (31), depending upon the accuracy of NT measurement. In the current survey, the mean NT was 1.61 mm for normal and 2.64 mm for DS cases (Table 1), which is similar to that for Asian populations in the normal group (32, 33), whereas the incidence of increased NT thickness was less than that reported in the results of two recent studies for DS (34, 35). Although NT alone is a rather good marker for DS screening, serum markers alone have a better detection rate (Table 2). Interestingly, the results of the current study show that a combination of NT and serum markers decreased the sensitivity of DS screening. This finding is consistent with those reported in previous studies (2, 9, 34).

Previous studies that the detection rate of DS screening based on the NT cut-off of 2.5 mm was 67.7% (9) and for the NT cut-off of 3 to 5 mm was 43% to 75% (17, 36). In the current study, sensitivity and specificity based on the NT cut-off of 1.89 mm was 77%.

It was also observed that the median MoM of PAPP-A decreased to 0.54 and β-hCG increased to 1.68 in DS pregnancies in the Iranian population, which is similar to results of a study of the Thai population (37). As previously reported, the median MOM values of PAPP-A and B-hCG in the Asian population were higher than for other populations (38). The current findings, however, showed that the median PAPP-A in the Iranian population was higher than for some Asian populations, such as the Chinese, Korean, Taiwanese and Vietnamese, whereas the median B-hCG was less than from these countries without weight correction (22).

The detection rate of PAPP-A and β-hCG (86% and 80%, respectively) with a 5% false positive rate obtained in the current study was comparable to those reported by Berktold et al (14). The present survey also showed that the combination of PAPP-A and β-hCG yielded a better detection rate than that reported by several centres (14, 39, 40).

This study was undertaken to design a novel decision-tree model based on serum markers for DS screening in the Iranian population. It revealed a better predictive value that achieved higher performance for DS detection in the first trimester. The importance of this model is that it promotes optimal selection in screening policies, in order to reduce unnecessary invasive testing.

The limitations of this study were its small sample size for DS pregnancies and the loss of a large number of samples during data collection and follow-up. Additionally, because Iran is a multi-ethnic country and considering the dependency of serum markers on ethnicity (41), further studies on various aspects of DS screening are highly recommended.

## Conclusion

The novel decision-tree model base on serum markers revealed a better predictive value to achieve high sensitivity and specificity of first trimester Down syndrome screening in Iranian population. Despite the large number of Down syndrome screening publications, the concentration of this study was skewed to improve it, based on the ethnicity and origin of the population studied. This study provides an effective model which is comparable to the results of other countries and it can also help clinicians in the accurate and reliable screening of Down syndrome and the right to choose parents for other stages of pregnancy. However, to increase the detection rate of the prediction algorithm and the performance of DS screening in the first trimester, the use of more serum markers and sonographic parameters is suggested.
